# Unusual Fragmentations of Silylated Polyfluoroalkyl Compounds Induced by Electron Ionization

**DOI:** 10.1021/jasms.5c00185

**Published:** 2025-08-12

**Authors:** Yufang Zheng, Edward P. Erisman, Weihua Ji, Stephen E. Stein, William E. Wallace

**Affiliations:** Mass Spectrometry Data Center, Biomolecular Measurement Division, 10833 National Institute of Standards and Technology, Gaithersburg, Maryland 20899, United States

**Keywords:** fluorinated alcohols, fluorinated carboxylic acids, ion fragmentation pathway, PFAS, reference library, reference spectrum

## Abstract

Per- and polyfluoroalkyl substances (PFAS) are environmentally persistent compounds that present analytical challenges due to their stability and low concentrations. In this study, electron ionization (EI) mass spectra of trimethylsilyl (TMS) derivatized fluorinated alcohols and carboxylic acids were examined to improve PFAS identification in the NIST Mass Spectral Reference Library. In contrast with the spectra of unsubstituted alcohol TMS compounds featuring losses of hydrocarbons, fluorinated alcohol TMS derivatives are characterized by the losses of fluorinated silyl groups. For example, a previously unreported [M–111]^+^ ion was consistently observed in compounds containing three methylene groups between the hydroxyl group and the first CF_2_ unit. Detailed quality assurance analysis using a suite of NIST software tools along with high-resolution TOF-MS confirmed the origin and elemental composition of these ions. MS^2^ experiments and full scan of TMS derivatives of fluorinated alcohols with varying numbers of methylene groups investigations suggest the formation of a five-membered ring intermediate as a key feature in this unique fragmentation pathway. These findings improve our understanding of PFAS fragmentation and support more accurate compound identification in analytical workflows.

## Graphical abstract

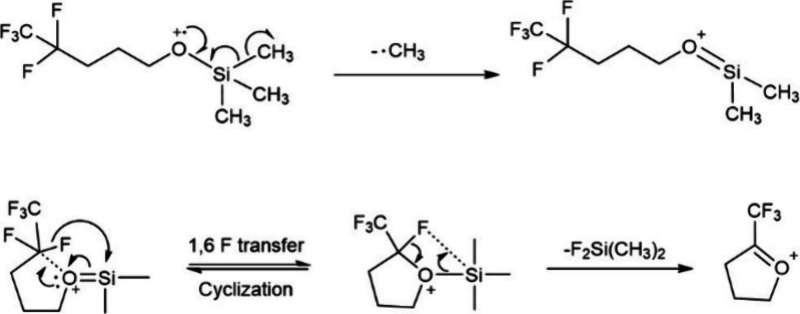

## Introduction

Per- and Polyfluoroalkyl Substances (PFAS), are environmental contaminants known for their chemical stability and resistance to decomposition. Sometimes called ″Forever Chemicals,″ their persistence allows them to accumulate in the environment over time. PFAS are found in many everyday items, including drinking water,^1^ air,^2,3^ food,^4^ and many consumer products.^5^ The associated health risks are thought to be significant, with studies linking PFAS exposure to cancer,^6^ liver damage,^7^ and ovarian dysfunction.^8^ Analyzing PFAS is particularly challenging due to their unique chemical structure and their presence in low concentrations in the environment.

The electron ionization of fluorocarbon compounds has been studied since the early days of mass spectrometry.^9–12^ As a result of this recent interest in PFAS identification, researchers have developed frameworks for confident PFAS identification, improving the reproducibility and standardization of environmental monitoring.^13–15^ Additionally, novel analytical methods using GC-MS have been introduced to study the thermal decomposition of PFAS and identify their breakdown products.^16^ Gas Chromatography–Mass Spectrometry (GC/MS) with silylated derivatives serves as a reliable and economical means of PFAS identification.^3,17,18^

The recent surge in interest in PFAS has motivated an effort to increase the coverage and verify the quality of all PFAS compounds in the NIST/EPA/NIH Mass Spectral Reference Library (NIST Standard Reference Database 1A).^19^ In the course of this work, we found some unexpected peaks in EI spectra of derivatized hydroxy-containing PFAS compounds. These corresponded to neutral losses of F_2_Si­(CH_3_)_3_ (109 Da), HOSi­(CH_3_)_3_F (111 Da) and others from molecular ions of certain TMS-derivatized fluorinated alcohols and carboxylic acids, particularly those bearing a fluorine atom five positions away from the silicon atom, which appear to enable a cyclization-driven fragmentation pathway. These losses are unusual compared with the fragmentation of their related nonfluorinated alcohols or carboxylic acids. A literature search produced no reports showing the losses of fluorinated silyl groups in these specific silylated PFAS compounds, such as loss of F_2_Si­(CH_3_)_3_ to form the [M-111]^+^ ion. The unexpected fragmentation was investigated by confirming its origin from authentic precursor and a reasonable fragmentation mechanism was proposed.

## Experimental Section

### Chemicals and Reagents

PFAS compounds used in this study were purchased from Sigma-Aldrich (Milwaukee, WI, USA) or SynQuest Laboratories, Inc. (Alachua, FL, USA) with reported purities exceeding 90%. Acetonitrile (anhydrous, 99.8%), chloroform, N,O-Bis­(trimethylsilyl) trifluoroacetamide (BSTFA) with 1% trimethylchlorosilane (TMCS), and pyridine (anhydrous, 99.8%) were obtained from Sigma-Aldrich.

### Sample Preparation

All PFAS standard solutions were prepared in acetonitrile or chloroform at a concentration of approximately 1 mg/mL. TMS derivatives were synthesized by mixing 50 μL of the sample solution with 50 μL of the BSTFA with 1% TMCS in 10 μL of pyridine. The reaction was carried out at 50 °C for 1 h.

### Instrumentation and Data Processing Software

A standard liquid injection method was carried out using an Agilent 8890–5977B GC/MSD system. A 1 μL liquid sample was directly injected into the inlet from an autosampler vial. Separation was achieved on a Restek Rxi-5Sil MS capillary column (30 m × 250 μm × 0.25 μm) with helium as the carrier gas at a constant flow rate of 1.2 mL/min and a split ratio of 50:1. A temperature-controlled cold injection system, CIS-5 (Gerstel), was used as the interface for subsequent GC/MS analysis. Cryogenic cooling of the CIS-5 from 150 °C and the oven from −10 °C was performed using liquid nitrogen for PFAS compounds with a retention index below 900. The inlet temperature was programmed to increase from 150 to 250 °C at 12 °C/min, with a 3 min hold at the final temperature. The oven temperature was initially ramped from −10 to 75 °C at 5 °C/min, followed by a second ramp from 75 to 200 °C at 20 °C/min. For PFAS compounds with a retention index above 900, the oven temperature was ramped from 35 °C (with a 10 min hold) to 270 °C at 15 °C/min, while the inlet temperature remained the same as previously described. The analysis was performed under electron ionization (EI) mode at 70 eV. Mass spectra were acquired in scan mode over a range of *m*/*z* 14–600. The ion source temperature was set to 230 °C, and the MS quadrupole temperature was maintained at 150 °C.

An Agilent Technologies 8890 GC, coupled with a JEOL AccuTOF GC Alpha mass spectrometer, was used to confirm the molecular formulas of unknown fragments observed in the EI spectra. The analysis used parameters same to those of the Agilent 8890–5977B GC/MS instrument for the GC component, with the exception of a 0.4-s MS recording interval, a 0.25-ns sampling interval, and a resolving power of 30,000 (fwhm) at *m*/*z* 614 during autotuning for the MS component. GC-MS^2^ product and precursor ion scans were performed using a triple quadrupole Agilent Technologies 7890*A*/7000A system, with the similar instrument settings as the Agilent 8890–5977B GC/MS. Nitrogen served as the collision gas, and collision voltages of 3, 5, 10, and 20 V were applied.

The EI mass spectra were extracted using the *NIST AMDIS* GC deconvolution program (version 2.73) for spectrum quality control.^20^ *NIST MS Interpreter* (version 3.4.5) was used to analyze ions in the spectra to confirm compound identity.^21^ The *NIST MS Search* (version 3.0) hybrid search algorithm, which compares spectra using direct and neutral loss peaks, was employed to identify unknown fragment ions in the spectra.^22,23^

## Results and Discussion

Structure of **Compound 1**: 4,4,5,5,5-pentafluoro-1-pentanol TMS derivative:

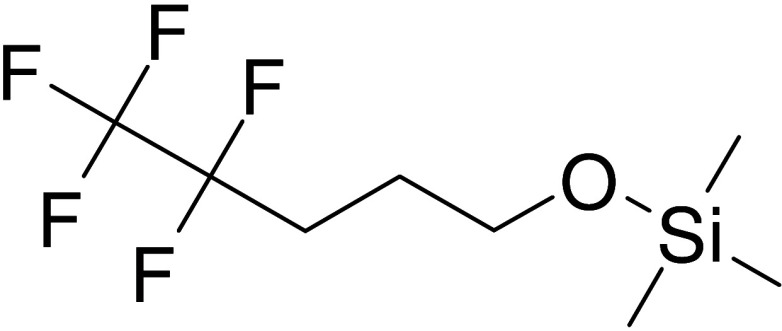



### Spectrum Comparison of TMS Derivatives of 1-Pentanol and 4,4,5,5,5-Pentafluoro-1-pentanol (Compound 1)

Polar alcohol compounds are often most conveniently identified by GC/MS following TMS-derivatization, as it increases both volatility and thermal stability, thereby enabling analysis under conventional liquid injection GC/MS conditions. It also provides richer fragmentation ions with higher abundances in the spectrum. The mass spectrum of silylated 1-pentanol is shown in Figure 1a. A prominent peak at *m*/*z* 145 corresponds to the loss of a methyl radical. Subsequent fragmentation involves the loss of propylene, butene, and pentene, resulting in peaks at *m*/*z* 103, 89, and 75, respectively, following the initial loss of a methyl radical ion at *m*/*z* 145. Fragmentation of silylated 1-pentanol is primarily characterized by losses of methyl and alkene groups, with the positive charge consistently localized on the oxygen or silicon atom. In contrast, the PFAS TMS derivative of 4,4,5,5,5-pentafluoro-1-pentanol **(compound 1)** displays distinctly different fragmentation behavior, as shown in Figure 1b. The near absence of M-15 peak indicates that the methyl-loss ion is very unstable. Major peaks at *m*/*z* 141, 139, 121, and 91 cannot be explained by the fragmentation pathways analogous to those observed in nonfluorinated analogues. Losses of silyl moieties predominate in the spectrum of **compound 1.**

**Figure 1:**
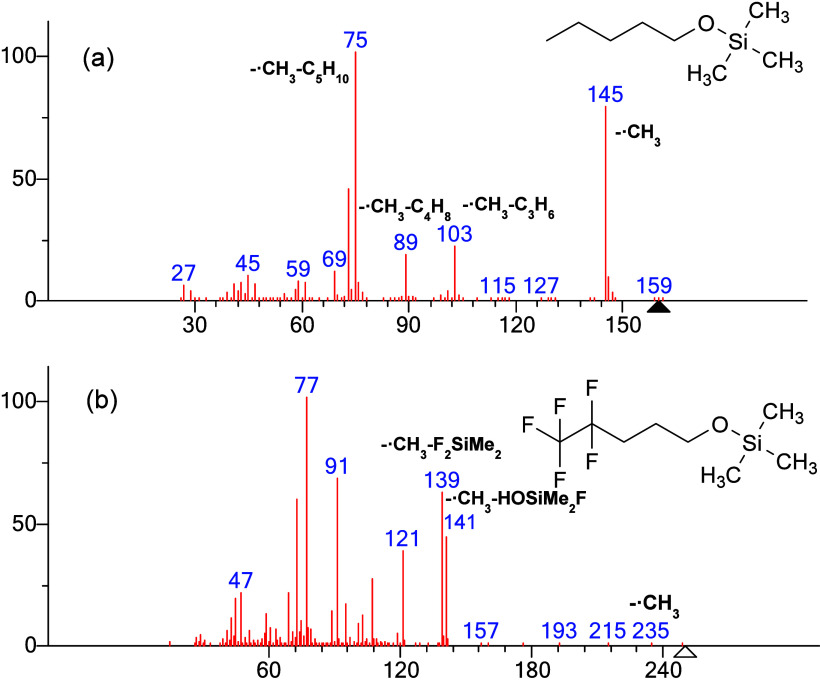
(a) EI spectrum of 1-pentanol TMS derivative, molecular ion at *m*/*z* 160. (b) EI spectrum of 4,4,5,5,5-pentafluoro-1-pentanol TMS derivative **(compound 1)**, molecular ion at *m*/*z* 250. Losses shown as chemical formulas.

### Spectrum Evaluation of Compound 1 by NIST MS Interpreter

*MS Interpreter* ^21^ was used to identify the ions in spectrum to confirm the identity of the compound. The use of this program can significantly accelerate the identification process and detect the errors that are difficult to identify manually. Figure 2 presents the mass spectrum of **compound 1** using *MS Interpreter*. The mass spectrum is displayed in the bottom panel. The middle panel shows the proposed fragment ion structure for the peak at *m*/*z* 121, with the fragment highlighted in red, losses indicated in black, and bond cleavages in green. The top panel lists possible molecular formulas corresponding to the mass value. Most of the product ions, as predicted by *MS Interpreter*, are marked in black on the spectrum indicating an ion in agreement with known fragmentation rules. The peak ion at *m*/*z* 121, marked in red, corresponds to an unusual loss, [M-(C_3_H_10_FOSi+HF)]^+^ as shown on the top panel. Furthermore, *MS interpreter* failed to predict a significant peak at *m*/*z* 139 marked in white. The mass difference between the ion and the molecular ion at *m*/*z* 250 is 111 Da. Other relatively small, unidentified white peaks were also observed.

**Figure 2:**
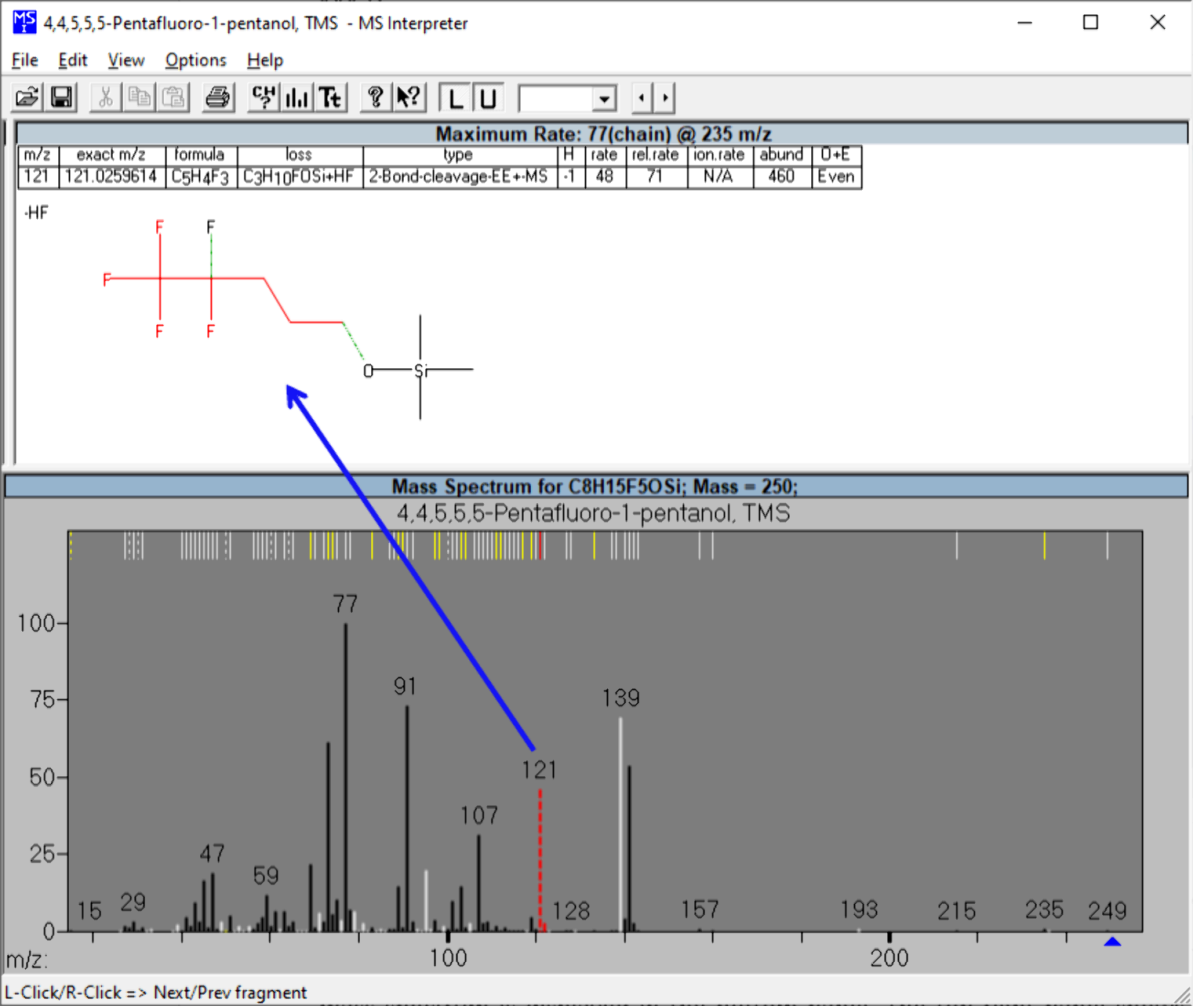
Electron ionization (EI) mass spectrum of **compound 1**, analyzed using *MS Interpreter*.

### Spectrum Evaluation by Hybrid Similarity Search

Since the structures of some PFAS TMS derivatives differ by a single, relatively inert group such as CF_2_, the hybrid similarity search method^22^ is particularly well-suited for identifying these derivatives based on spectra of these similar compounds. To better understand the origin of the *m*/*z* 139 ion, the hybrid search method was employed by searching against the 2023 NIST/EPA/NIH EI Mass Spectral Reference Library. The hybrid search uses neutral loss in its scoring. A head-to-tail comparison with a very high match factor of 914 between **compound 1** and the mass spectrum of the TMS derivative of 4,4,5,5,6,6,6-heptafluoro-hexanol (molar mass 300) shifted by 50 Da (corresponding to the difference in CF_2_ groups between compound 1 and 4,4,5,5,6,6,6-heptafluoro-hexanol) both contain the fragment at *m*/*z* 139 indicating the loss of 111 Da is common to TMS derivatized PFAS compounds (Figure 3). This is further supported by the presence of *m*/*z* 189 in the TMS derivative of 4,4,5,5,6,6,6-heptafluoro-hexanol, corresponding to [M-111]^+^ and a DeltaMass^22^ difference of 50 Da, which corresponds to a loss of CF_2_, representing the mass difference between the query and library compounds.

**Figure 3:**
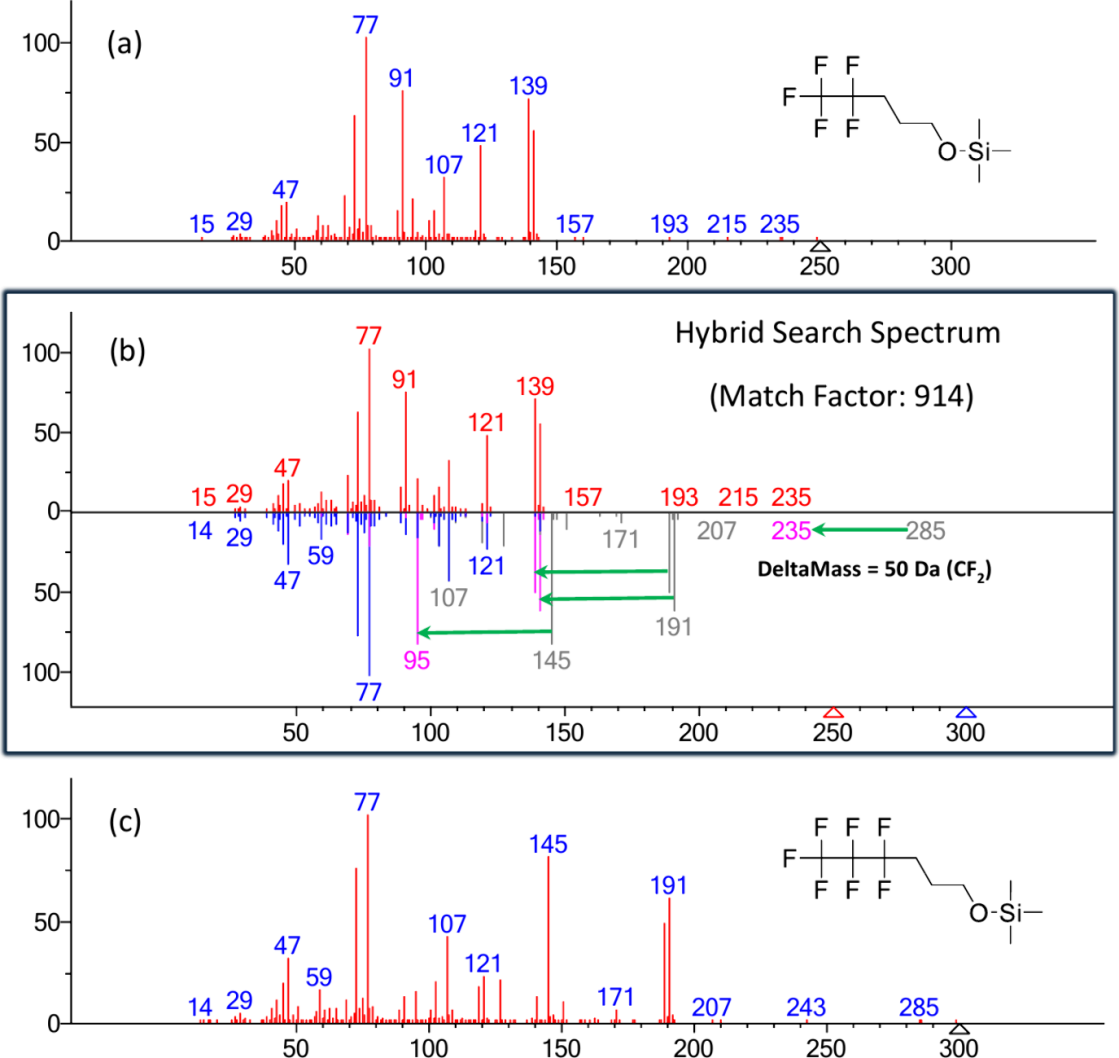
Hybrid search comparison between compound 1 and the TMS derivative of 4,4,5,5,6,6,6-heptafluorohexanol. (a) Mass spectrum of compound 1. (b) Head-to-tail comparison between the mass spectrum of compound 1 (top, red peaks) and the hybrid search result (bottom). The hybrid spectrum consists of blue, gray, and magenta peaks. The blue and gray peaks originate from the mass spectrum of the TMS derivative of 4,4,5,5,6,6,6-heptafluorohexanol. The magenta peaks represent ions shifted by a mass difference (DeltaMass) of 50 Da, corresponding to a CF_2_ group. (c) Mass spectrum of the TMS derivative of 4,4,5,5,6,6,6-heptafluorohexanol.

### High Resolution Analysis and Product Ion Scans of Compound 1

To confirm the elemental composition of the *m*/*z* 139 ion, a JEOL high-resolution TOF mass spectrometer was used to measure the spectrum of **compound 1** shown in Figure 4. Accurate mass measurement and isotopic pattern give a *m*/*z* value of 139.0365, corresponding to a formula C_5_H_6_F_3_O^+^ with mass accuracy <1 ppm. The C_5_H_6_F_3_O^+^ ion is formed by loss of C_3_H_9_F_2_Si• from the molecular ion of compound **1**, where C_3_H_9_F_2_Si• can be assigned as the result of sequential fragmentation involving the loss of •CH_3_ followed by F_2_SiMe_2_. The *m*/*z* 77.0217 ion representing FSiMe_2_^+^ is the most abundant ion, which strongly suggests fluorine atom can easily migrate to silicon atom. The peak at *m*/*z* 141.0321 arises from the loss of C_3_H_10_FOSi• from the molecular ion of **compound 1**, corresponding to losses of •CH_3,_ followed by the loss of FSiMe_2_OH. In order to lose FSiMe_2_OH, one F atom migrates to the silicon atom, and one hydrogen atom transfers to oxygen atom. Hydrogen transfer to the oxygen atom in the fragmentation of primary alcohol TMS derivative had been well documented.^24^ Notably, the *m*/*z* 107.0323 ion (CH_2_=O^+^–SiMe_2_F) is formed via a classic α-cleavage of **compound 1** following methyl loss and fluorine migration to the silicon atom.

**Figure 4:**
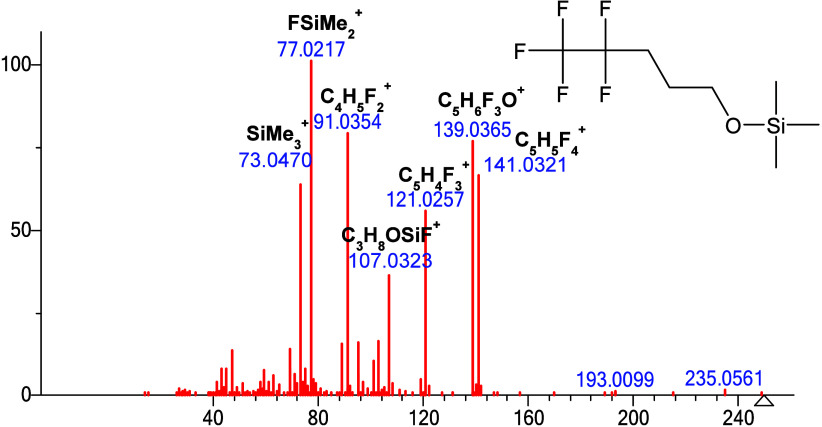
High resolution EI spectrum of **compound 1**, molecular ion at *m*/*z* 250.0807­(<1 ppm). Ions are labeled with the molecular formula of the fragment.

To further elucidate the additional peaks observed in the spectrum of **compound 1**, product ion scan experiments were conducted by GC/MS^2^ for the *m*/*z* 139 and 141 ions. The product ion scan of *m*/*z* 139 generates peaks at *m*/*z* 91 due to the loss of CHOF, as well as *m*/*z* 71 and *m*/*z* 69, as shown in Figure 5, while the *m*/*z* 141 ion generates fragment peaks at *m*/*z* 121 due to the loss of HF, *m*/*z* 101, and *m*/*z* 95, as shown in Figure 6.

**Figure 5:**
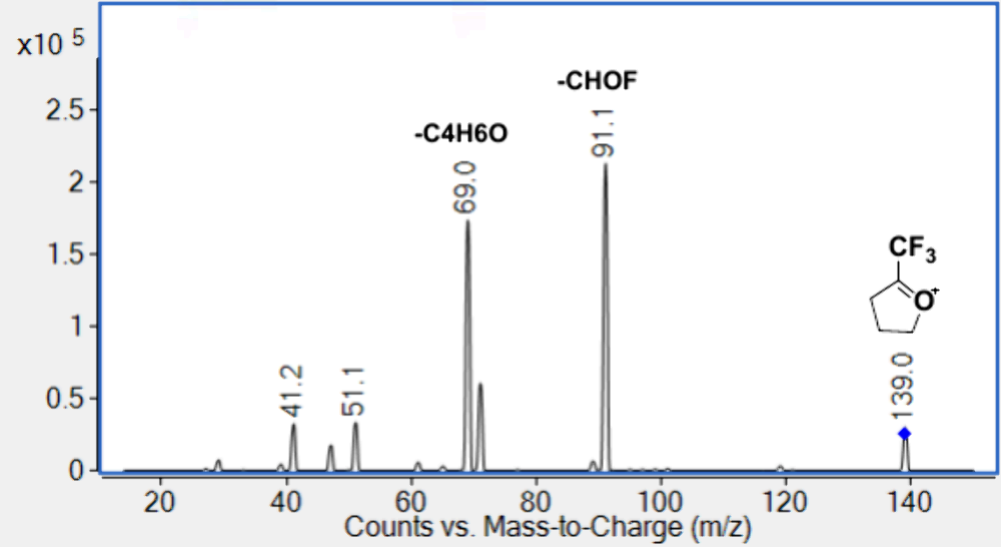
GC-MS^2^ product ion scan of compound 1 at *m*/*z* 139.

**Figure 6:**
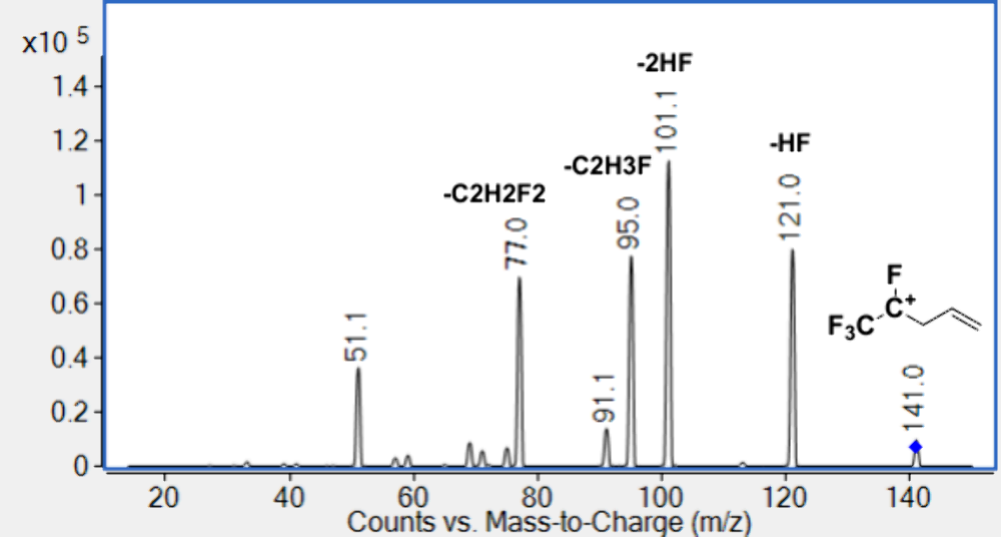
GC-MS^2^ product ion scan of compound 1 at *m*/*z* 141.

Overall, fragmentations of PFAS alcohol TMS derivatives behave differently compared with common alcohol TMS derivatives. F transfer to the silicon atom and subsequent losses of silyl moiety dominate in the fragmentations.

### Effect of the Number of Methylene Groups (CH_2_)_n_ on the Fragmentation of TMS Derivatized PFAS Alcohols

A series of TMS derivatives of fluorinated alcohols with varying numbers of methylene groups were analyzed using the JEOL high-resolution TOF mass spectrometer to investigate the ion [M–111]^+^. The spectrum of **compound 1**, which has three methylene groups, was included in Figure 7b for comparison purpose and the prominent peak at *m*/*z* 139.0365 corresponds to the [M–111]^+^ ion. Interestingly, the [M–111]^+^ peak at *m*/*z* 153.0523 (Figure 7c) is significantly less intense in the spectrum of the TMS derivative of 5,5,6,6,6-pentafluorohexan-1-ol (four methylene groups), and in the spectrum of 3,3,4,4,4-pentafluoro-1-butanol TMS (two methylene groups), the corresponding [M–111]^+^ peak at *m*/*z* 125.0211 (Figure 7a) is barely detectable. This observation suggests that fragmentation behavior is strongly influenced by the length of the methylene (CH_2_) chain. The presence of three CH_2_ units between the oxygen atom and the nearest CF_2_ group appears to be necessary for this fragmentation. Only PFAS compounds with this spacing undergo the distinctive loss of 111 Da, suggesting the possible formation of a five-membered ring following methyl loss.

**Figure 7:**
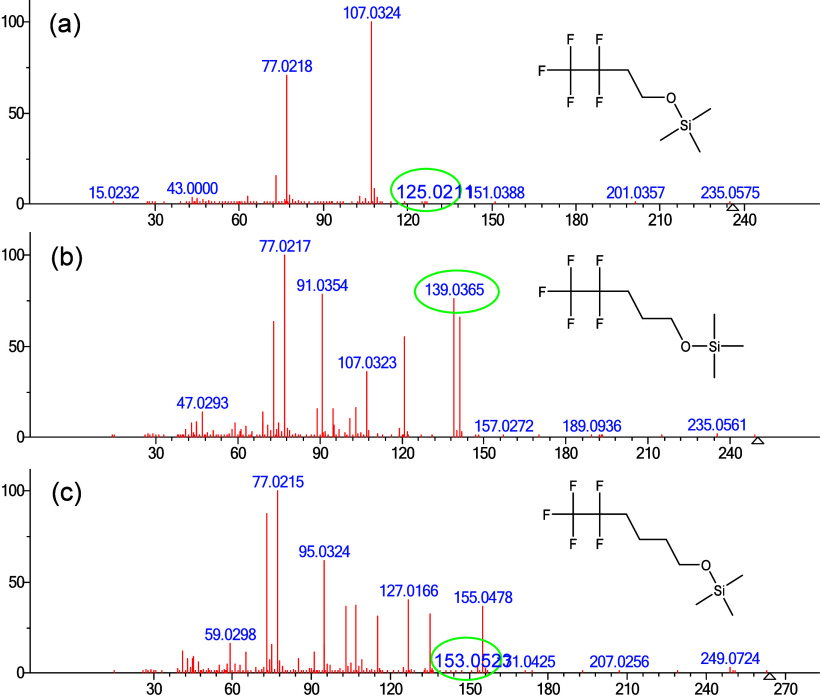
High Resolution EI mass spectra of TMS derivatives of fluorinated alcohols with varying numbers of CH_2_ groups with the [M-111]^+^ ion circled in green. (a) TMS derivative of 3,3,4,4,4-pentafluoro-1-butanol, (b) TMS derivative of 4,4,5,5,5-pentafluoro-1-pentanol (compound 1), and (c) TMS derivative of 5,5,6,6,6-pentafluorohexan-1-ol. The open triangle on the mass axis indicates the molecular ion.

### Effects of the Number of Fluorine Atoms on the Fragmentation of TMS Derivatized PFAS Alcohols

With the CH_2_ chain length fixed at three methylene groups, a series of TMS derivatives of fluorinated alcohols with varying numbers of fluorine atoms were analyzed to investigate the formation of the [M–111]^+^ ion. The results reveal that high-intensity [M–111]^+^ peaks are consistently observed across these fluorinated alcohol compounds. In Figure 8a, the spectrum corresponds to the TMS derivative of 4,4,4-trifluoro-1-butanol, featuring a terminal CF_3_ group and producing a prominent peak at *m*/*z* 89. The spectrum of compound 1, containing a CF_3_CF_2_ group, is shown in Figure 8b for comparison. The bottom spectrum in Figure 8c, representing the TMS derivative of 4,4,5,5,6,6,6-heptafluoro-1-hexanol with a longer CF_3_CF_2_CF_2_ chain, also shows a pronounced [M–111]^+^ peak at *m*/*z* 189. Notably, as the molecular weight of these compounds increases by increments of 50 Da, the *m*/*z* values of the [M–111]^+^ fragments also increase by 50 Da, indicating a consistent fragmentation pattern correlated with the length of the fluorinated chain.

**Figure 8:**
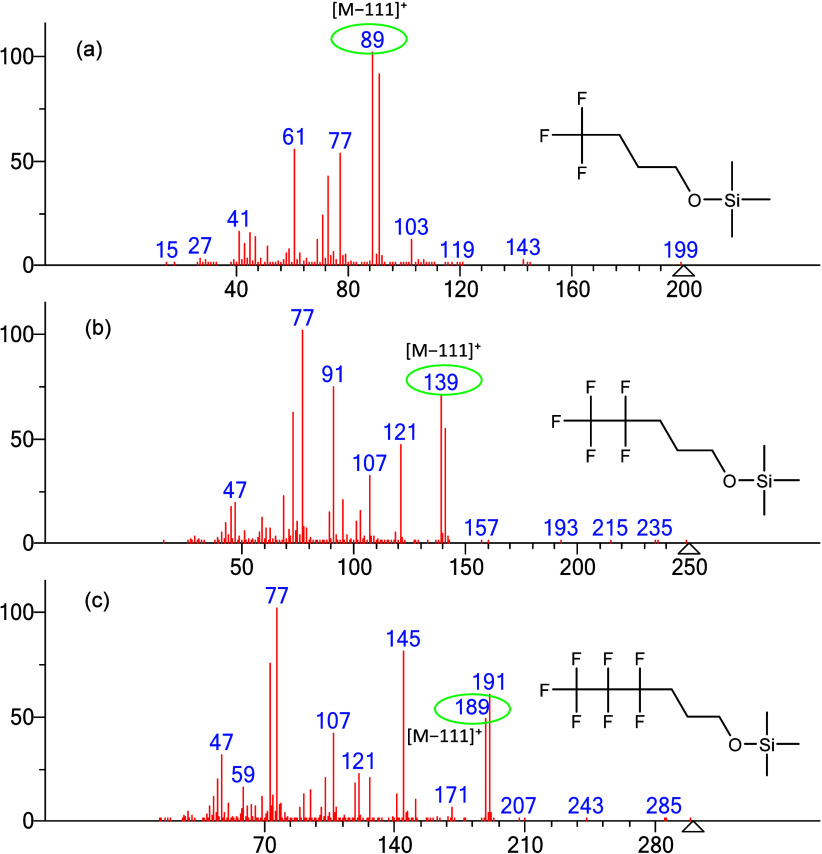
EI mass spectra of fluorinated alcohols as their TMS derivatives, showing spectra for compounds with increasing numbers of fluorine atoms with the [M-111]^+^ ion circled in green: (a) TMS derivative of 4,4,4-trifluoro-1-butanol, (b) TMS derivative of 4,4,5,5,5-pentafluoro-1-pentanol (**compound 1**), and (c) TMS derivative of 4,4,5,5,6,6,6-heptafluoro-1-hexanol.

### Proposed Fragmentation Mechanism for the Formation of [M-111] Cation

Fragmentation of **compound 1** is characterized by the loss of fluorinated silyl groups. Scheme 1 proposes a mechanism for the formation of the *m*/*z* 139 ion and its related fragment ion.

**Scheme 1:**
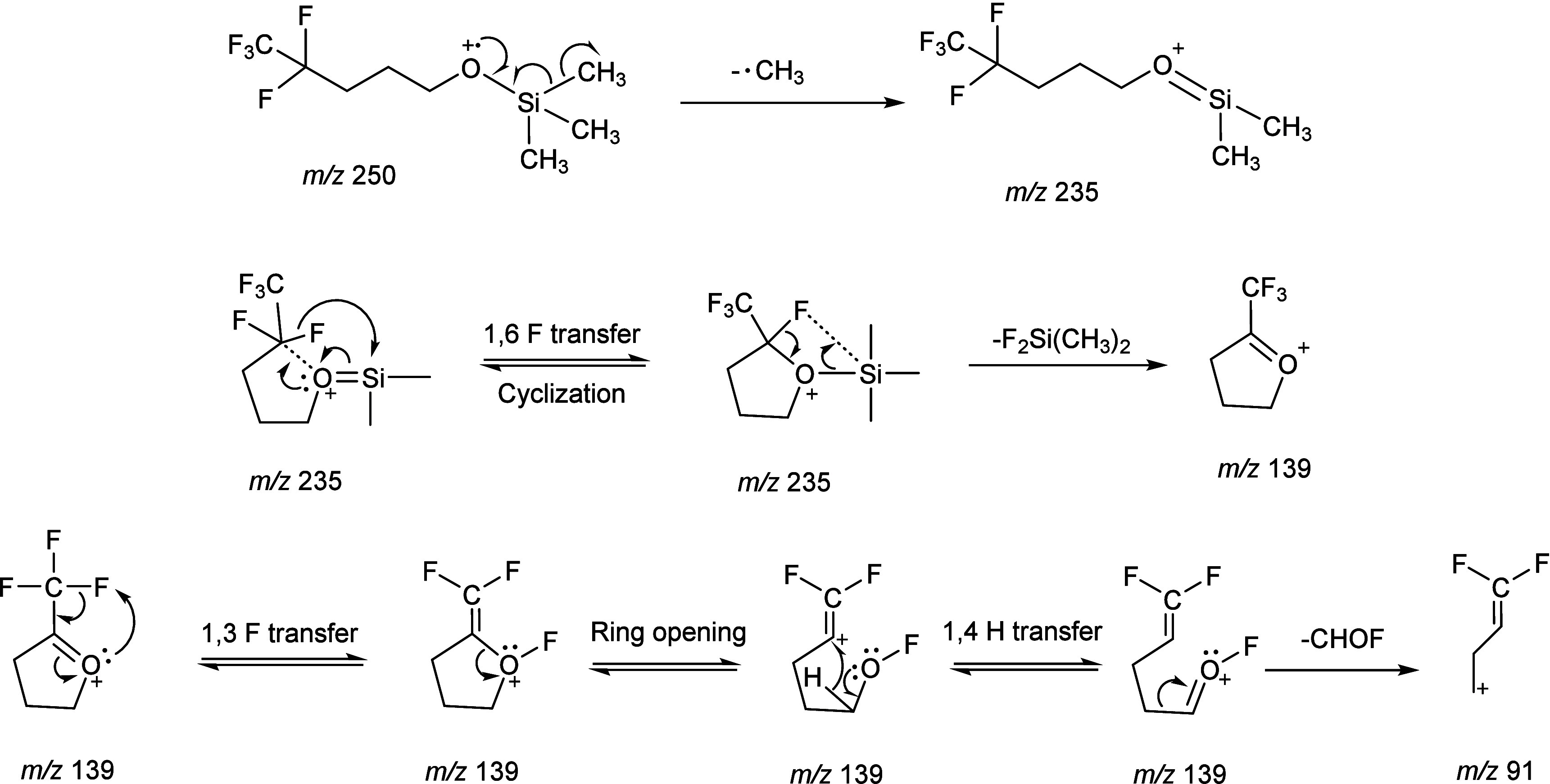
Proposed Fragmentation Pathway for the Formation of the *m/z* 139 from Molecular Ion of **Compound 1.**

Methyl loss is a well-known pathway in the fragmentation of alcohol TMS derivatives and the loss usually produces a major product ion as it does from the TMS derivatives of most other derivatized groups.^24^ In the mass spectrum of **compound 1**, the methyl-loss peak at *m*/*z* 235 is relatively small. A similar pattern appears in the mass spectra of the TMS derivatives of 3,3,4,4,4-pentafluoro-1-butanol (two CH_2_ groups) and 5,5,6,6,6-pentafluorohexan-1-ol (four CH_2_ groups), indicating that the methyl-loss ion is generally unstable and likely undergoes further fragmentation.

Diekman et al.^25^ reported in the TMS derivative of a primary alcohol, a hydrogen atom migrates to the silicon atom after methyl loss, giving a characteristic ion at *m*/*z* 89 as CH_2_=O^+^-Si­(CH_3_)_2_H. Similarly, we proposed 1,6-fluorine transfer to the silicon atom as shown in Scheme 1. The driving force of fluorine atom transfer from carbon to silicon is thermodynamically favorable because the Si–F bond (576.4 ± 17 kJ/mol) is significantly stronger than the C–F bond (513.8 ± 10.0 kJ/mol).^26^ The formation of a stronger Si–F bond results in a net release of energy. This leads to the stabilization of silicon via formation of a strong Si–F bond. Moreover, the most abundant ion in the EI spectrum of **compound 1** is assigned as FSiMe_2_ at *m*/*z* 77, which conclusively shows the F atom migration.^27^

As shown in Figure 7, formation of the **[M-111]^+^** ion requires the presence of three CH_2_ units. Initially, methyl loss occurs. This is followed by fluorine atom migration, leading to the formation of a stable five-membered ring ion, in which the positive charge is localized on the oxygen atom rather than the carbon. This species undergoes a neutral loss of F_2_SiMe_2_ via an elimination reaction, generating the *m*/*z* 139 ion. A subsequent neutral loss of CHOF leads to the formation of the *m*/*z* 91 ion. The formation of the five-membered ring is an intermediate for stabilizing the fragmentation process, which explains the consistent observation of the [M-111]^+^ ion across various fluorinated compounds.

### Extending the Unusual Fragmentation Pathway to Fluorinated Carboxylic Acid TMS Derivatives

Results for derivatized carboxylic acids are similar to those of derivatized alcohols discussed above when there are three carbons between the oxygen and the first CF_2_ group. As shown in Figure 9, the corresponding [M–111]^+^ ions appear for the TMS derivatives of heptafluorohexanoic acid, nonafluoroheptanoic acid, and pentadecafluorodecanoic acid, respectively. With each compound increasing in molecular weight by 50 Da, the [M–111]^+^ ions also shift by 50 Da.

**Figure 9:**
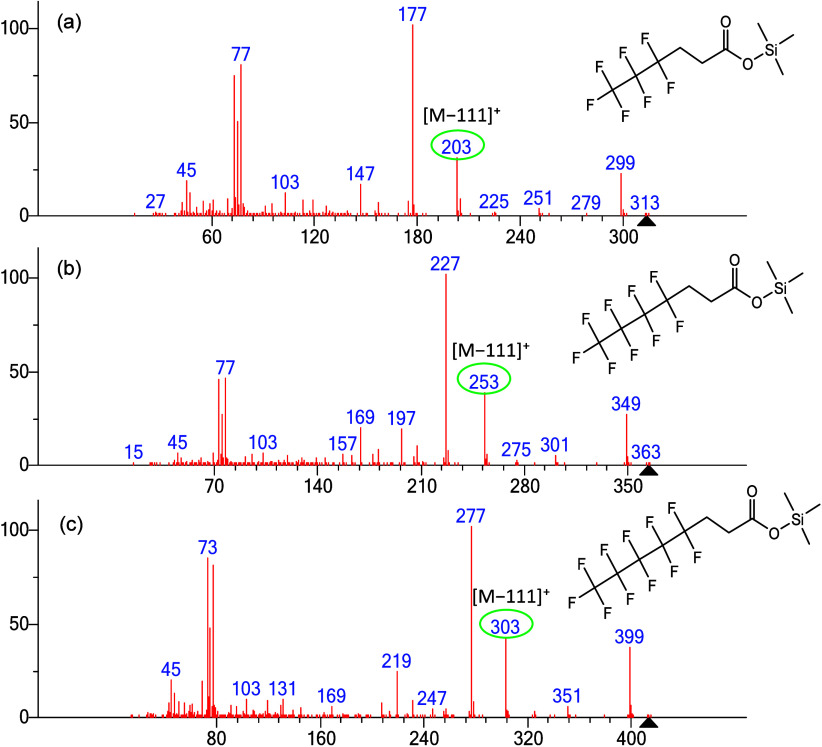
EI mass spectra of TMS derivatives of fluorinated carboxylic acids with varying numbers of fluorine atoms with the [M-111]^+^ ion circled in green. (a) TMS derivative of 4,4,5,5,6,6,6-heptafluorohexanoic acid, (b) TMS derivative of 4,4,5,5,6,6,7,7,7-nonafluoroheptanoic acid, and (c) TMS derivative of 4,4,5,5,6,6,7,7,8,8,9,9,10,10,10-pentadecafluorodecanoic acid.

## Conclusions

In the course of improving the coverage and qualilty of PFAS spectra in NIST/EPA/NIH Mass Spectral Reference Library, it was noted that some TMS-derivatized fluorinated alcohols and carboxylic acids demonstrate unusual fragmentation patterns such as producing an unexpected major fragment ion [M–111]^+^. While this behavior does not depend on the number of fluorine atoms, it is influenced by their location—specifically, the presence of a fluorine atom five positions away from the silicon atom appears necessary to enable the proposed cyclization and subsequent fragmentation pathway. Software tools, including *MS Interpreter* and the hybrid similarity search, confirmed ion peaks, while high-resolution mass spectrometry validated the chemical formula of this [M–111]^+^ ion. The hypothesis of a five-membered ring intermediate is proposed for the pathway of this unusual, major fragment ion. These findings not only improve the understanding of PFAS-related fragmentation mechanisms but also provide a framework for identifying structurally related compounds. The results aid in more precise identification and differentiation of PFAS derivatives in analytical applications.
